# Detection of ***β***-Lactamases and Outer Membrane Porins among *Klebsiella pneumoniae* Strains Isolated in Iran

**DOI:** 10.1155/2014/726179

**Published:** 2014-08-03

**Authors:** Ali Hashemi, Fatemeh Fallah, Soroor Erfanimanesh, Parastu Hamedani, Shadi Alimehr, Hossein Goudarzi

**Affiliations:** ^1^Department of Microbiology, Shahid Beheshti University of Medical Sciences, P.O. Box 19857-17443, Tehran, Iran; ^2^Pediatric Infectious Research Center, Mofid Children Hospital, Shahid Beheshti University of Medical Sciences, Tehran, Iran

## Abstract

This descriptive study was accomplished on 83* K. pneumoniae* strains isolated from two hospitals in Tehran, Iran. Antibiotic susceptibility tests were performed by disc diffusion and broth microdilution methods. ESBLs, MBL, Amp-C, and KPC producing strains were detected by phenotypic confirmatory test, combination disk diffusion test (CDDT), Amp-C detection kit, and modified Hodge test, respectively. OXA-48, NDM-1, and CTX-M-15 genes were detected by PCR and sequencing methods. The outer membrane porins such as OmpK35 and OmpK36 were analysed by SDS-PAGE, PCR, and sequencing methods. From 83* K. pneumoniae* isolates, 48 (57.5%), 3 (3.5%), 23 (28%), and 5 (6%) were ESBL, MBL, Amp-C, and KPC positive, respectively. The CTX-M-15 gene was detected in 30 (62.5%) and OXA-48 gene was found in 2 (4.1%) of the 48 ESBL-producing isolates. Two isolates harboured both OXA-48 and CTX-M-15; NDM-1 gene was not detected in this study. Outer membrane porin, OmpK35, was detected in 30 (62.5%) of 48 ESBL-producing isolates while OmpK36 was found in 35 (72.91%) of 48 ESBL-producing isolates. In this study, fosfomycin and tigecycline were more effective than other antibiotics. The high prevalence of *β*-lactamase-producing* K. pneumoniae* detected in this study is of great concern, which requires infection control measures including antibacterial management and identification of *β*-lactamases-producing isolates.

## 1. Introduction

Carbapenems currently represent the antibiotics of choice for treatment of serious infections caused by multidrug-resistant strains of* K. pneumoniae* which produce extended-spectrum *β*-lactamases (ESBLs) [1]. Carbapenem resistance in* K. pneumoniae* is based on various mechanisms that may involve carbapenemase production, upregulation of efflux pumps, or loss of porins, including OmpK35, OmpK36, and OmpK37. Some commonly detected carbapenemases are* Klebsiella pneumoniae *carbapenemase(KPC), New Delhi metallo-*β*-lactamase-1 (NDM-1), and OXA-48-type enzymes whose respective genes are located on plasmids that enable their transfer between different gram-negative bacteria species [2]. The Ambler class D *β*-lactamase OXA-48, which hydrolyzes penicillins and imipenem and extended-spectrum cephalosporins, was first reported from a carbapenem-resistant* K. pneumoniae* isolate from Turkey. Outbreaks of OXA-48-producing* K. pneumoniae* and other isolates have been reported from various regions of Turkey and once in the United Kingdom. Subsequently, single isolates of OXA-48-producing* K. pneumoniae* have been reported from Belgium, Lebanon, the United Kingdom, Morocco, Tunisia, Argentina, and India [3]. Emergence of NDM-1 has been considered as a global threat because bacteria which possess this metallo-*β*-lactamase are resistant to almost all *β*-lactam antibiotics, aminoglycosides, fluoroquinolones, and other classes of antibiotics. The rapid emergence of NDM-1 may be due to transmissible plasmids which can spread among different isolates; subsequently, NDM-1 can spread throughout the world. The bla_NDM-1_ gene is carried on different plasmids and can harbour a large number of resistance genes such as KPC, OXA-48, and CTX-M-15 [4]. KPC-producing isolates of* K. pneumoniae* and other bacteria have spread rapidly throughout the world. KPC-producing* K. pneumoniae* isolates have been reported from Iran and mortality rate among patients with positive KPC was 33% [5]. The other reports have been described in other areas, including China, Demark, Greece, Hungary, Argentina, Brazil, Colombia, Finland, France, Germany, Italy, Sweden, Norway, Puerto Rico, and the United Kingdom [6]. KPC-producing strains typically possess ESBLs or Amp-C enzymes, which can hydrolyze carbapenems, penicillins, cephalosporins, and aztreonam [4]. In* K. pneumoniae*, two major porins, OmpK36 and OmpK35, are homologous to OmpF and OmpC, respectively. Clinically, most of the ESBL-producing* K. pneumoniae* strains express only OmpK36, whereas the majority of* K. pneumoniae* that do not produce ESBLs express both OmpK36 and OmpK35. The absence of OmpK35 may be one of the factors contributing to the antibiotic resistance of ESBL-producing* K. pneumoniae*. Reports also revealed that OmpK36 may play an important role in carbapenem resistance of* K. pneumoniae* strains that produce ESBL or Amp-C-type *β*-lactamases [7]. The aims of this study were the phenotypic detection of *β*-lactamases and OXA-48, NDM-1, and CTX-M-15 genes among* K. pneumoniae *strains isolated in Iran. The detection of outer membrane porins in ESBL-producing* K. pneumoniae* strains is also analysed.

## 2. Materials and Methods

### 2.1. Bacterial Isolates

From October 2011 to May 2012, 83 nonduplicate nonconsecutive* K. pneumoniae* from males 27 (32.53%), females 14 (16.86%), and infants 42 (50.60%) were collected from hospitalized patients at Taleghani and Mofid Children Hospitals, Tehran, Iran. The isolates were stored at −20°C in trypticase soy broth containing 20% glycerol.

### 2.2. Antimicrobial Susceptibility Testing

Antimicrobial susceptibility of each* K. pneumoniae* isolate was determined by the Kirby-Bauer disk diffusion method (Mast Group, Merseyside, UK) on Mueller-Hinton agar (Merck, Germany). The results were then interpreted as recommended by Clinical Laboratory Standards Institute (CLSI) [8] or FDA breakpoints (tigecycline) guidelines. Disks of penicillins (piperacillin (PIP, 100 *μ*g), ampicillin (AMP, 10 *μ*g)), *β*-lactam/*β*-lactamase inhibitor combinations (piperacillin/tazobactam (PTZ, 100/10 *μ*g)), cephems (ceftazidime (CAZ, 30 *μ*g), cefotaxime (CTX, 30 *μ*g), cefepime (FEP, 30 *μ*g), ceftriaxone (CRO, 30 *μ*g), and cefpodoxime (CPD, 30 *μ*g)), monobactams (aztreonam (ATM, 30 *μ*g)), carbapenems (imipenem (IPM, 10 *μ*g), meropenem (MEM, 10 *μ*g), doripenem (DOR, 10 *μ*g), and ertapenem (ETP, 10 *μ*g)), aminoglycosides (gentamicin (GEN, 10 *μ*g), amikacin (AK, 30 *μ*g)), tetracyclines (tetracycline (TE, 10 *μ*g)), fluoroquinolones (ciprofloxacin (CIP, 5 *μ*g)), inhibitors (trimethoprim-sulfamethoxazole (TS, 2.5 *μ*g)), fosfomycins (fosfomycin/trometamol (FOT, 200 *μ*g)), and tigecycline (TGC, 15 *μ*g) were used and* Escherichia coli* ATCC25922 was used as a control strain.

### 2.3. Minimum Inhibitory Concentration (MIC)

Antimicrobial susceptibility was determined by broth microdilution method according to the guidelines of CLSI. Briefly, microbial inoculums in Mueller-Hinton broth were adjusted to final concentration of 0.5 on the McFarland scale and were diluted to 1 : 20. 10 microliters of each inoculum was added to wells containing 100-microliter Muller-Hinton broth and the following antibiotics: imipenem, meropenem, cefepime, ampicillin, piperacillin/tazobactam, cefotaxime, ceftriaxone, and ceftazidime (GLAXO England Co. and Himedia). After 24-hour incubation at 37°C microbial growth for each treatment was evaluated. Results are shown in Table 2.

### 2.4. Phenotypic Detection of *β*-Lactamases

Combination disk diffusion test (CDDT) was performed for presumptive identification of MBLs by imipenem, meropenem, doripenem, and ertapenem (Mast Group, Merseyside, UK) alone and in combination with EDTA [9]. Detection of ESBLs was tested for all the isolates by combination disk diffusion test (CDDT) containing ceftazidime (CAZ) and cefotaxime (CTX) with CAZ 30 *μ*g + CA 10 *μ*g and CTX 30 *μ*g + CA 10 *μ*g per disc (Mast Group, Merseyside, UK). The zones of inhibition were compared for the CTX, CAZ discs with that of the CAZ 30 *μ*g + clavulanic acid (CA) 10 *μ*g and CTX 30 *μ*g + CA 10 *μ*g disc. An increase in zone diameter of ≥5 mm in the presence of clavulanic acid was interpreted positive for the presence of ESBL in the test organism.* Escherichia coli* ATCC25922 and* K. pneumoniae* ATCC700603 were used as negative and positive controls for ESBL production, respectively. Presumptive identification of KPC enzyme was performed for all the* K. pneumoniae* isolates by modified Hodge test [7]. Amp-C was detected using the D69C AmpC Detection kit developed by Mast Group [10].

### 2.5. Detection of Resistance Genes

Plasmids DNA were extracted by Plasmid Mini Extraction Kit (Bioneer Company, Korea). *β*-lactamases genes were amplified by PCR. Amplification for CTX-M-15 gene was performed with the following primers: CTX-M-15-F (5′-GCGATGGGCAGTACCAGTAA-3′) and CTX-M-15-R (5′-TTACCCAGCGTCAGATTCCG-3′). Amplification was carried out with the flowing thermal cycling conditions: 5 min at 94°C and 36 cycles of amplification consisting of 1 min at 94°C, 1 min at 55°C, and 1 min at 72°C, with 5 min at 72°C for the final extension. DNA fragments were analysed by electrophoresis in a 1% agarose gel at 95 V for 45 min in 1X TBE containing ethidium bromide.

### 2.6. Sequencing

The PCR purification kit (Bioneer Company, Korea) was used to purify PCR products. Sequencing of forward strand was also performed by Bioneer Company (Korea). BLAST in NCBI, Chromas 1.45, and MEGA-4 software were used to analyse the nucleotide sequences.

### 2.7. Outer Membrane Porins Analysis

The outer membrane porins OmpK35 and OmpK36 were analysed by sodium dodecyl sulphate polyacrylamide gel electrophoresis (SDS-PAGE) using membrane extracts. PCR and sequencing methods were carried out to confirm the presence of the outer membrane genes [11].

## 3. Results

A total of 83* K. pneumoniae* strains were isolated from patients. 45 (54.21%) of the isolates were from patients at Taleghani Hospital and 38 (45.79%) others were from Mofid Children Hospital. The patients comprised 27 (32.53%) males, 14 (16.86%) females, and 42 (50.60%) infants. The studied strains were isolated from the following wards: pediatrics, 38 (45.7%); outpatient, 13 (15.6%); intensive care units (ICUs), 9 (10.8%); surgery, 7 (8.4%); NICU, 4 (4.8%); bone marrow transplant unit, 3 (3.6%); hematology, 2 (2.4%); endocrine, 2 (2.4%); gastrology, 2 (2.4%); orthopedics, 1 (1.2%); and other wards, 2 (2.4%). Bacterial isolates were recovered from different clinical specimens: urine, 33 (39.7%); blood culture, 27 (32.5%); wound, 7 (8.4%); sputum, 7 (8.4%); intra-abdominal, 4 (4.8.%); cerebrospinal fluid, 1 (1.2%); and other samples, 4 (4.8%). Antimicrobial drug-resistance patterns of the 83* K. pneumoniae* isolates are shown in Table 1. MIC results for the studied isolates are shown in Table 2. The combination disk diffusion test (CDDT) was applied to test ESBL production in the isolates using ceftazidime and cefotaxime alone and in combination with clavulanic acid. The results showed that 48 (57.5%) of the isolates were positive for ESBL production of which 28 (62.3%) belonged to Taleghani Hospital and 20 (52.7%) to Mofid Children Hospital. Using the same method, 3 (3.5%) of the 83 isolates of* K. pneumoniae* were MBL positive. Amp-C screening by Mast Group kit showed that 23 (28%) of the isolates were Amp-C positive of which 14 (31.3%) were isolated from Taleghani Hospital and 9 (23.7%) from Mofid Children Hospital. Modified Hodge test (MHT) was applied to detect KPC production. The results showed that 5 (6%) of the isolates were KPC positive of which 3 (6.7%) were isolated from Taleghani and 2 (5.3%) from Mofid Children Hospital. Number and percent of *β*-lactamases present in* K. pneumoniae* isolates are shown in Table 3. The CTX-M-15 gene was detected in 30 (62.5%) of 48 ESBL-producing* K. pneumoniae* isolates while OXA-48 gene was found in 2 (4.1%) of the 48 ESBL-producing isolates. In this study, NDM-1 gene was not detected. Two isolates harboured both OXA-48 and CTX-M-15. Outer membrane porin, OmpK35, was detected in 30 (62.5%) of the 48 ESBL-producing isolates while OmpK36 was found in 35 (72.91%) of the 48 ESBL-producing isolates.

## 4. Discussion

Recent studies indicated that emergence of antibiotic-resistant clinical isolates and multidrug-resistant* K. pneumoniae* strains have been increased among patients in Iran [12]. The lowest rates of resistance in isolates were observed for fosfomycin 3 (3.6%), tigecycline 5 (6.02%), amikacin 12 (14.4%), ertapenem 21 (25.3%), doripenem 20 (24%), meropenem 20 (24%), imipenem 20 (24%), and piperacillin/tazobactam 22 (26.5%). The highest rates of resistance observed belonged to ampicillin 65 (78.3%), cefpodoxime 57 (68.6%), cefotaxime 50 (60.2%), piperacillin 50 (60.2%), ceftriaxone 49 (59%), aztreonam 49 (59%), ciprofloxacin 46 (55.5%), and ceftazidime 46 (55.5%). So, the best coverage against the study isolates was obtained with tigecycline and fosfomycin. Another concerned problem is related to their multidrug resistance which restricts the treatment procedure. Resistance to antibiotics is related to various enzymes that are produced, including ESBLs, MBLs, KPC, and Amp-C which belong to Ambler A, B, and C groups [13, 14]. The high rate of ESBLs among hospitalized patients is a global problem. The prevalence of ESBL-producing isolates of* K. pneumoniae* varies in different countries [15]. In this study, 48 (57.5%) of* K. pneumoniae* were identified as ESBL producers by phenotypic tests, which was less than Ahangarzadeh et al.'s study 124 (83.2%) and Feizabadi et al.'s study 75 (72.1%) in Iran [16, 17] and another study in Turkey (60%) and was less than Latin America (45.4%), Western Pacific (24.6%), and Europe (22.6%) [15]. The high rate of ESBL prevalence in Iran and its widespread dissemination is a cause of worry.In Spain, the results revealed that 133 adults and 29 pediatric patients were infected with ESBL-producing* K. pneumoniae* [18]. In this study, 24 adults and 33 pediatric patients were infected with ESBL-producing* K. pneumoniae. *The wide use of drugs might facilitate the spread of antibiotic resistance; thus control of *β*-lactamases especially ESBL-producing* K. pneumoniae *within infant units should be considered as precedence. Resistance to carbapenem is related to MBLs and KPC *β*-lactamases which belong to Ambler A and B groups [19]. In this study by using the CDDT method, 3 (3.5%)* K. pneumoniae* isolates were identified as MBL producers. Also, 5 (6%) of* K. pneumoniae* isolates were identified as KPC producers by MHT, which was less than Shahcheraghi et al.'s study 11 (47.8%) in Iran and Chen et. al's study 75 (72.1%) in China [6, 20]. The prevalence of Amp-C in Iran and worldwide is unknown, due to laboratories having a problem in accurately detecting this resistance mechanism. In this study by using the D69C AmpC Detection kit, 23 (28%)* K. pneumoniae* isolates were identified as Amp-C producers. Rodriguez-Bano et al. showed that 100 patients were infected with plasmid-mediated Amp-C *β*-lactamases including 17 Amp-C-producing* K. pneumoniae* [21]. In another study, Thean et al. reported that 127 (49.8%) of the test isolates (*Proteus, E. coli, *and* K. pneumoniae*) were Amp-C positive [22]. The particular CTX-M enzyme type in ESBL-producing* K. pneumoniae* varies geographically. CTX-M-15 enzyme, which belongs to the CTX-M-1 group, is the most prevalent CTX-M allele with a worldwide distribution [23]. In this study, the CTX-M-15 gene was detected in 30 (62.5%) of 48 ESBL-producing isolates and all were resistant to ceftazidime and cefotaxime. Feizabadi et al. in Iran showed that 46.51% (*n* = 40) of* K. pneumoniae* isolates were *bla*_CTX-M_ positive [24]. Also, Goudarzi et al. reported that 74 (74%) of* Escherichia coli* isolates were *bla*_CTX-M-15_ positive [25]. Reports of OXA-48-producing Enterobacteriaceae have recently dramatically increased worldwide, particularly in the Middle East and Europe. Potron et al. showed two enterobacterial isolates harbouring *bla*_OXA-48_ gene encoded by a novel Tn1999 transposon derivation that additionally contains the ESBL gene *bla*_CTX-M-15_ [26]. In a recent study, two isolates harboured both OXA-48 and CTX-M-15.* K. pneumoniae* produces two major porins, OmpK35 and OmpK36. However, most ESBL-expressing* K*.* pneumoniae* clinical isolates produce only OmpK36 porin [27]. OmpK35 and OmpK36 provide a channel that allows a wide range of antibiotics to penetrate to cell wall. We have reported the existence of two major porins, OmpK36 and OmpK35, in* K. pneumoniae*. OmpK35 was detected in 30 (62.5%) of the 48 ESBL-producing isolates while OmpK36 was found in 35 (72.91%) of the 48 ESBL-producing isolates. Loss of this porin may be one of the factors contributing to antimicrobial resistance among ESBL-producing* K. pneumoniae* and may favour the selection of additional mechanisms of resistance. Microbiology laboratories must be able to identify resistant bacteria in a timely suitable manner, especially those that are falsely susceptible* in vitro* to antibiotics that may be considered for therapy of infected adults and infants. Bacteriological excellence is needed more than ever, and it is vital that ESBLs enzymes, Amp-C, and carbapenemases be promptly and accurately detected.

## Figures and Tables

**Table 1:** Antimicrobial susceptibility testing results of 83 isolates of *K. pneumoniae* collected from Mofid Children and Taleghani Hospitals, Tehran, Iran.

Antibiotic	Resistant number (%)	Intermediate number (%)	Sensitive number (%)
Aztreonam (10 *μ*g)	49 (59%)	3 (3.6%)	31 (37.3%)
Meropenem (10 *μ*g)	20 (24%)	2 (2.4%)	61 (73.5%)
Gentamicin (10 *μ*g)	29 (35%)	3 (3.6%)	51 (61.5%)
Ciprofloxacin (30 *μ*g)	46 (55.5%)	4 (4.8%)	33 (39.7%)
Amikacin (30 *μ*g)	12 (14.4%)	4 (4.8%)	67 (80.7%)
Ceftazidime (30 *μ*g)	46 (55.4%)	3 (3.6%)	34 (41%)
Imipenem (10 *μ*g)	20 (24%)	2 (2.4%)	61 (73.5%)
Cefotaxime (30 *μ*g)	50 (60.2%)	2 (2.4%)	31 (37.3%)
Cefepime (FEP, 30 *μ*g)	30 (36.15%)	9 (10.8%)	44 (53.05%)
Tetracycline (TE, 10 *μ*g)	33 (39.7%)	3 (3.6%)	57 (68.6%)
Ampicillin (AMP, 10 *μ*g)	65 (78.3%)	2 (2.4%)	16 (19.2%)
Piperacillin (PIP, 100 *μ*g)	50 (60.2%)	0 (0.0%)	33 (39.7%)
Ceftriaxone (CRO, 30 *μ*g)	49 (59%)	2 (2.4%)	32 (38.5%)
Cefpodoxime (CPD, 30 *μ*g)	57 (68.6%)	3 (3.6%)	23 (27.7%)
Tigecycline (TGC, 15 *μ*g)	5 (6.02%)	25 (30.1%)	53 (63.8%)
Doripenem (DOR, 10 *μ*g)	20 (24%)	1 (1.2%)	62 (74.6%)
Ertapenem (ETP, 10 *μ*g)	21 (25.3%)	2 (2.4%)	57 (68.6%)
Piperacillin/tazobactam (PTZ, 100/10 *μ*g)	22 (26.5%)	5 (6.02%)	56 (67.4%)
Fosfomycin/trometamol (FOT, 200 *μ*g)	3 (3.6%)	12 (14.4%)	68 (82%)

**Table 2:** Minimum inhibitory concentration of different antibiotics against 83 *K. pneumoniae* isolates.

Antibiotics	MIC (*µ*g/mL)		
	Range	50%	90%
Meropenem	0.25–256	1	32
Imipenem	0.25–256	1	16
Ceftazidime	1–>256	64	>256
Ceftriaxone	0.5–>256	16	>256
Cefepime	0.5–>256	16	>256
Cefotaxime	0.5–>256	16	>256
Piperacillin/tazobactam	0.25–256	4	128
Ampicillin	2–>256	256	>256

**Table 3:** Number and percent of *β*-lactamases present in *K. pneumoniae* collected from Mofid Children and Taleghani Hospitals, Tehran, Iran.

*β*-Lactamases content	Taleghani number (%)	Mofid Children number (%)	Total number (%)
ESBL	28 (62.3%)	20 (52.7%)	48 (57.5%)
MBL	2 (4.4%)	1 (2.6%)	3 (3.5%)
Amp-C	14 (31.3%)	9 (23.7%)	23 (28%)
KPC	3 (6.7%)	2 (5.3%)	5 (6%)
ESBL + MBL	2 (4.4%)	1 (2.6%)	3 (4%)
ESBL + Amp-C	11 (24.4%)	12 (31.6%)	23 (28%)
ESBL + KPC	3 (6.7%)	2 (5.3%)	5 (6%)
ESBL + MBL + Amp-C	2 (4.4%)	1 (2.6%)	3 (3.5%)
ESBL + Amp-C + KPC	2 (4.4%)	1 (2.6%)	3 (3.5%)
ESBL + MBL + KPC	0 (0%)	0 (0%)	0 (0%)
ESBL + MBL + KPC + Amp-C	0 (0%)	0 (0%)	0 (0%)
